# Meloxicam Inhibited the Proliferation of LPS-Stimulated Bovine Endometrial Epithelial Cells Through Wnt/β-Catenin and PI3K/AKT Pathways

**DOI:** 10.3389/fvets.2021.637707

**Published:** 2021-07-09

**Authors:** Luying Cui, Yang Qu, Hele Cai, Heng Wang, Junsheng Dong, Jun Li, Chen Qian, Jianji Li

**Affiliations:** ^1^College of Veterinary Medicine, Yangzhou University, Jiangsu Co-innovation Center for Prevention and Control of Important Animal Infectious Diseases and Zoonoses, Yangzhou, China; ^2^Joint International Research Laboratory of Agriculture and Agriproduct Safety of the Ministry of Education, Yangzhou, China

**Keywords:** meloxicam, bovine endometrial epithelial cells, proliferation, lipopolysaccharide, Wnt/β-catenin, PI3K/AKT

## Abstract

Meloxicam is a non-steroidal anti-inflammatory drug and has been used to relieve pain and control inflammation in cows with metritis and endometritis. Meloxicam has been found to be effective in inhibiting tissue or cell growth when it is used as an anti-inflammatory therapy. However, the influence of meloxicam on bovine endometrial regeneration has not been reported. This study was to research the effect of meloxicam (0.5 and 5 μM) on the proliferation of primary bovine endometrial epithelial cells (BEECs) stimulated by *Escherichia coli* lipopolysaccharide. The cell viability, cell cycle, and cell proliferation were evaluated by Cell Counting Kit-8, flow cytometry, and cell scratch test, respectively. The mRNA transcriptions of prostaglandin-endoperoxide synthase 1 (*PTGS1*) and *PTGS2*, Toll-like receptor 4, and proliferation factors were detected using quantitative reverse-transcription polymerase chain reaction. The activations of phosphatidylinositol 3-kinase (PI3K)/protein kinase B (AKT) and Wnt/β-catenin pathways were determined using western blot and immunofluorescence. As a result, co-treatment of meloxicam and lipopolysaccharide inhibited (*P* < 0.05) the cell cycle progression and reduced (*P* < 0.05) the cell healing rate and the mRNA level of proliferation factors as compared with the cells treated with lipopolysaccharide alone. Meloxicam decreased (*P* < 0.05) the lipopolysaccharide-induced *PTGS2* gene expression. Neither lipopolysaccharide nor meloxicam changed *PTGS1* mRNA abundance (*P* > 0.05). Meloxicam inhibited (*P* < 0.05) the lipopolysaccharide-activated Wnt/β-catenin pathway by reducing (*P* < 0.05) the protein levels of β-catenin, c-Myc, cyclin D1, and glycogen synthase kinase-3β and prevented the lipopolysaccharide-induced β-catenin from entering the nucleus. Meloxicam suppressed (*P* < 0.05) the phosphorylation of PI3K and AKT. In conclusion, meloxicam alone did not influence the cell cycle progression or the cell proliferation in BEEC but caused cell cycle arrest and inhibited cell proliferation in lipopolysaccharide-stimulated BEEC. This inhibitory effect of meloxicam was probably mediated by Wnt/β-catenin and PI3K/AKT pathways.

## Introduction

Post-partum uterine infection is a common reproductive disease in cattle, causing an increased culling rate and huge economic losses to the breeding industry. Bovine endometritis is a common disease occurring in post-partum cows, with an incidence rate of 10–30% (1). Infections of the uterus with *Escherichia coli* precede infection by other common pathogenic bacteria via the production of lipopolysaccharide (LPS) (2, 3).

Following calving, the uterine involution includes tissue repair, endometrial regeneration, and bacteria elimination (4). The formation of new epithelium will be important in maintaining the next-round pregnancy and in re-establishing the innate defense system (5). Bovine endometrial epithelial cells (BEECs) are required in defending against *E. coli* and in repairing the epithelium (6). The vascular endothelial growth factor (VEGF) has been found to promote endometrial repair in mice and primates (7). The connective tissue growth factor (CTGF) participates in endometrial repair and has many physiological functions such as promoting angiogenesis, mitosis, and cell adhesion (8). The insulin-like growth factor and insulin-like growth factor receptor (IGFR) participate in the regulation of mitosis of endometrial epithelial cells (9). The transforming growth factor-β (TGF-β) is involved in the differentiation and proliferation of many kinds of cells, initiating tissue repair (10).

The Wnt pathway is a highly conserved signal transduction cascade that regulates cell growth and proliferation. In primates and mice, the Wnt/β-catenin pathway is involved in endometrial repair (11, 12). The activation of the adenomatous polyposis coli/axin/glycogen synthase kinase-3β/β-catenin/casein kinase 1 complex results in the dephosphorylation of β-catenin, which then enters the nucleus and activates downstream c-Myc, cyclin D1, and VEGF transcription to regulate cell cycle and cell proliferation (13, 14). The phosphatidylinositol 3-kinase (PI3K)/protein kinase B (AKT) signal transduction pathway participates in cell growth, proliferation, and differentiation. It has been proved that the PI3K/AKT pathway is involved in endometrial repair in human and dairy goats (15, 16).

The conventional treatment for uterine infection includes environmental disinfection, uterine irrigation, and uterine infusion with large amounts of antibiotics. Non-steroidal anti-inflammatory drugs (NSAIDs) in combination with antibiotics are used increasingly in the treatment of metritis and endometritis (17). Studies have shown that NSAIDs provide therapeutic effects such as analgesia, ovarian function recovery, and prevention and treatment of uterine inflammation (18). Meloxicam (MEL) is an NSAID that preferentially inhibits cyclooxygenase-2 (COX-2) in most animals, but this affinity has not been verified in dairy cows (19). MEL has been found to decrease the viability of breast cells in cows with mastitis, suggesting a potential side effect of MEL to bovine breast tissue (20). More experimental trials and clinical reports are required to clarify the mechanism and effect of MEL in treating bovine post-partum uterine diseases. So far, there are few studies regarding the effect of MEL on the survival and proliferation of BEEC.

The goal of this study was to reveal the influence and mechanism of MEL on BEEC proliferation. The BEEC was treated with LPS. The changes in the cell cycle, cell scratch test, the mRNA transcriptions of prostaglandin-endoperoxide synthase 1 (*PTGS1*) and *PTGS2*, Toll-like receptor 4 (*TLR4*), and growth factors and the key proteins of Wnt/β-catenin and PI3K/AKT pathways were determined.

## Materials and Methods

### Culture of BEECs

The cells were isolated as described from Dong et al. (21). Briefly, cows with no signs of genital disease or microbial infection were selected in the slaughterhouse, based on the presence of foul smell, characteristic visual appearance, and vaginal discharge (1). The uterus was collected aseptically and stored on ice at 4°C until further treatment in the laboratory. The uterine horn was cut into 3–4-cm-long tissue blocks and was washed with PBS (pH value from 7.2 to 7.4). Then the tissue blocks were transferred to 0.1% streptose (P5147, Sigma, USA) diluted with DMEM/F-12 (D8900, Sigma, USA) and digested at 4°C for 16–20 h. Under sterile conditions, the tissue block was removed, and the endometrial tissue was scraped with a sterilized scalpel. The cell suspension was centrifuged at 100 × *g* for 5 min and washed with PBS three times. The cells were then collected and resuspended with DMEM/F-12 containing 15% fetal bovine serum (FBS, Gibco, USA) and 50 U/ml penicillin/streptomycin, and inoculated into a 25-cm^2^ bottle. The cells were cultured in the incubator at 37°C and 5% CO_2_ saturation humidity. The medium was changed every 1–2 days. The primary cells could be obtained after 3–4 days.

### MEL Treatment

MEL (M3935, Sigma-Aldrich, USA) was dissolved in DMSO and stored at −20°C. It has been reported that the injection dose of 0.5 mg/kg MEL could maintain the blood concentration of 0.2 μg/ml MEL in cows with endometritis (22). In addition, MEL was used in a bovine lymphocyte test (23, 24), which provided references for MEL concentration in this experiment. In this study, two concentrations of MEL, 0.5 and 5 μM, were selected. First, 100 mg MEL was dissolved in 26.782 ml DMSO. Then, a 1 ml DMSO–MEL solution was added in 49 ml DMEM/F-12 for storage at −20°C. This storage concentration was 200 μM. MEL was filtered before use. The experiment was divided into four groups: the control group, the LPS group, the MEL group, and the LPS plus MEL co-treatment (LPS-MEL) groups. According to the results of previous reports in our lab, 10 μg/mL LPS (L2630, Sigma, USA) did not influence the cell viability of BEEC (25). Therefore, 10 μg/ml LPS was selected for this study.

### Cell Viability Assay

The Cell Counting Kit-8 (CCK-8, Dojindo Molecular Technologies, Inc., Japan) was used to determine the effect of MEL and LPS on cell viability. The cells were seeded on 96-well plates with a density of 1 × 10^3^ cells per well and grew to 80% fusion in a 5% CO_2_ incubator at 37°C. Then the cells were treated with 5 × 10^−2^, 5 × 10^−1^, 5, or 5 × 10^1^ μM MEL or with 10 μg/ml LPS. CCK-8 was added into each well and incubated at 37°C for 2–4 h. The optical density was then read at 450 nm using a microplate (Tecan, Austria).

### Cell Cycling Analysis

BEEC was treated with MEL or LPS or LPS–MEL for 24 h. The cells were washed twice with cold PBS and fixed with 70% ethanol at 4°C for 12–24 h. Then the cells were washed twice with cold PBS and resuspended with RNase A and propidium iodide (C1052, Beyotime, China) in the dark at 37°C for 30 min. Cell cycle was detected by flow cytometry (LSRFortessa, BD Biosciences, USA) and was analyzed by FlowJo software 7.6 (Becton, Dickinson, and Company, Ashland, USA) to obtain the relative number of cells in each phase of the cell cycle.

### Cell Scratch Test

Before cell culture, three parallel lines, each 0.5 cm apart, were drawn on the back of a six-well plate using a marker pen. The cells were then seeded on this plate with a density of 1 × 10^6^ cells per well and were cultured overnight in a cell incubator with 5% CO_2_ at 37°C. The cell scratch test started when the cells reached 90% fusion. The cells were scratched by using the tip of a 200 μl sterilized pipette. This scratching line was perpendicular to the previous three lines and was along the diameter of the well. The cells were quickly washed with PBS at least three times until no cell was visible at the scratch microscopically. Then the DMEM/F-12 containing LPS or MEL was added to the well. The cell treatments were as follows: LPS, MEL (0.5 or 5 μM), or LPS–MEL (0.5 or 5 μM).

**Figure 1:**
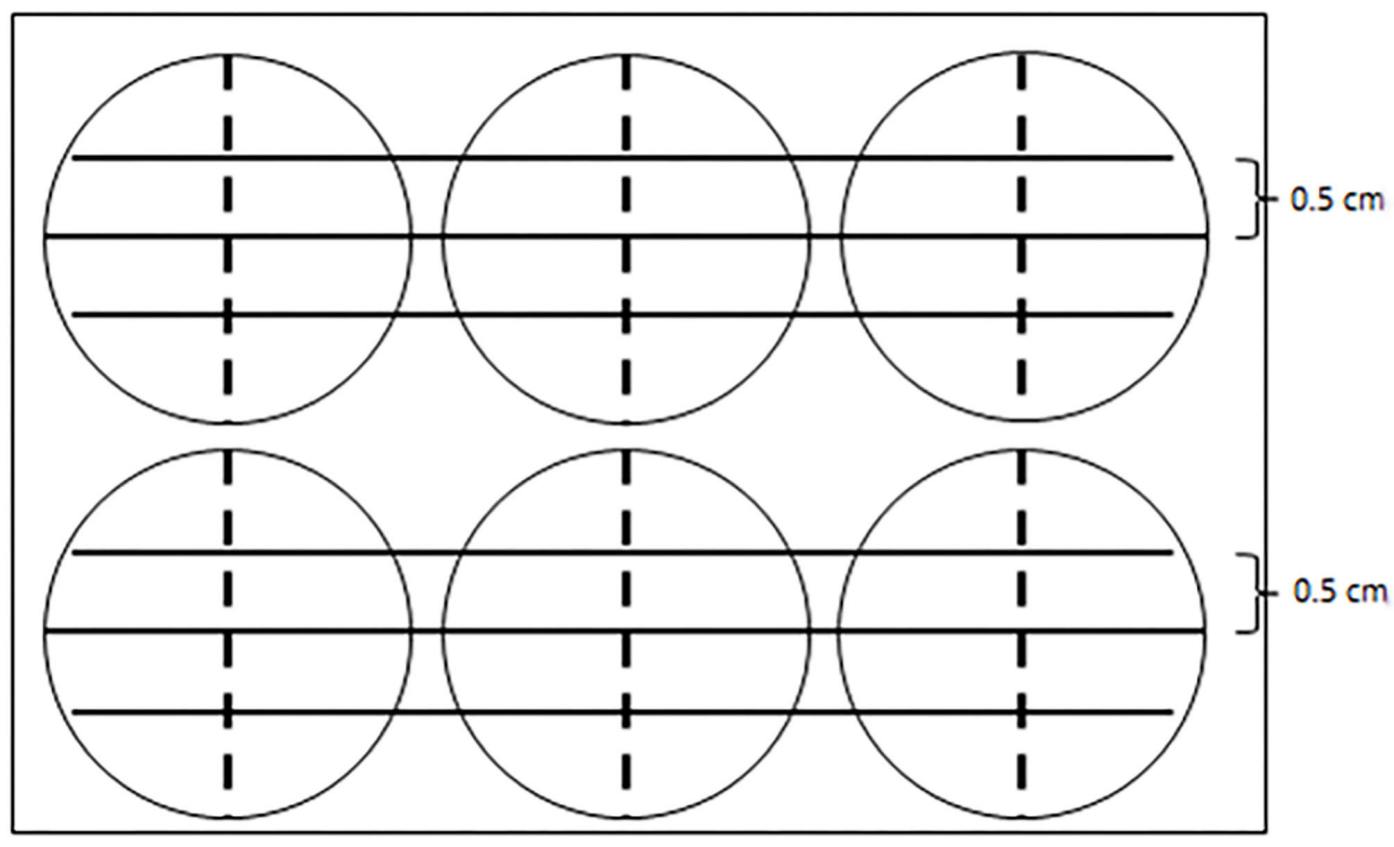
A diagram showing the reference line in the cell scratch test. Three parallel solid lines were drawn by a marker pen on the back of a six-well plate. Each parallel line was 5 cm apart. The tip of a 200-μl sterilized pipette scratched along the dotted line, which was perpendicular to the solid lines and was along the diameter of each well.

The six-well plate was observed under the inverted microscope at ×100 magnification. Photos were taken immediately after the scratching (0 h) and at 3, 6, 12, and 24 h at fixed positions, which were localized with the help of the three parallel lines (Figure 1). The photos were analyzed by Image-Pro Plus software 6.0 (Media Cybernetics, Rockville, USA). The healing rate of the scratch reflects cell proliferation. The calculations were as follows:

Scratch width = scratch area/scratch length
Healing rate = (scratch width at 0 h – scratch width at various time points)/scratch width at 0 h × 100%

### RNA Extraction and Quantitative Reverse-Transcription Polymerase Chain Reaction

The cells were seeded into a six-well plate and were treated with LPS, MEL (0.5 and 5 μM), or LPS–MEL (0.5 and 5 μM) for 0, 3, 12, and 24 h. Total RNA was extracted using TRIzol reagent (ET111, TRAN, China) according to the manufacturer's instruction. A Nanodrop 2000 spectrophotometer (Thermo, USA) was used to detect the quantity and purity of the RNA. The absorption ratio (A260/A280) was determined to be between 1.8 and 2.1. The total RNA was reverse-transcribed into cDNA by a PrimeScript RT regent kit gDNA eraser (DRR047A, Takara, Japan). The cycle conditions were as follows: 95°C for 2 min; 95°C for 5 s and 60°C for 34 s, 40 times; 95°C for 15 s; 60°C for 5 s; 60–95°C, 0.5°C gradient heating. The reaction system included 10 μl SYBR Premix Ex Taq™ II (RR820A, TaKaRa, Japan), 1 μl of each primer, 1 μl cDNA template in a final volume of 20 μl per reaction. The 2^−Δ*ΔCT*^ method was used to calculate the relative abundances of mRNA transcripts (26). Each group was repeated three times. The β-actin (*ACTB*) was used as the internal control. The primers were designed according to the mRNA sequences of bovine *ACTB, PTGS1, PTGS2, TLR4, VEGFA*, cellular communication network factor 2 (*CCN2*), insulin-like growth factor 1 receptor (*IGF1R*), *TGFB1*, and *TGFB3* published in GenBank. A single product was amplified by each primer pair. All the polymerase chain reaction (PCR) products were purified and sequenced (TsingKe Biotech, Beijing, China), and the sequence results were analyzed using BLAST and compared to the GenBank database (http://blast.ncbi.nlm.nih.gov/blast.cgi). The primer sequences are shown in Table 1.

**Table 1:** The list of primer sequences.

**Gene**	**Forward primers**	**Reverse primer**	**Product size (bp)**	**Accession number**
*ACTB*	CATCACCATCGGCAATGAGC	AGCACCGTGTTGGCGTAGAG	156	NM_173979.3
*PTGS1*	TGCCATCCGAACTCCATCTT	AATGTGCTTGCCTTCCAGTAC	116	NM_001105323.1
*PTGS2*	GCACAAATCTGATGTTTGCATTC	AGCTGGTCCTCGTTCAAAATCT	80	NM_174445.2
*TLR4*	GCTCTGCCTTCACTACAGGGACT	CTGGGACACCACGACAATAACC	106	NM_174198.6
*VEGFA*	GACCCTGGTGGACATCTTCC	CACACAGGGCACACACTCC	127	NM_001316992.1
*CCN2*	AGCTGACCTGGAGGAGAACA	GTCTGTGCACACTCCGCAGA	139	NM_174030.2
*IGF1R*	TTAAAATGGCCAGAACCTGAG	ATTATAACCAAGCCTCCCAC	314	NM_001244612.1
*TGFB1*	CGAGCCCTGGACACCAACTA	AGGCAGAAATTGGCGTGGTA	137	NM_001166068.1
*TGFB3*	CTGTGCGTGAATGGCTCTTG	CATCATCGCTGTCCACACCT	153	NM_001101183.1

### Western Blot Analysis

BEECs were treated with LPS, MEL (0.5 and 5 μM), or LPS–MEL (0.5 and 5 μM). The total protein was extracted and quantified by a bicinchoninic acid protein assay kit (P0010, Beyotime, China). The protein (20–30 μg) was separated by 10% SDS–polyacrylamide gels and was transferred to the polyvinylidene difluoride (PVDF) membrane (Millipore, Germany). The PVDF membrane was incubated in 5% skimmed milk diluted by TBST (0.05% Tween-20 Tris-HCl buffer) to prevent its binding to non-specific protein. The primary antibody used for incubation included β-catenin, c-Myc, cyclin D1, glycogen synthase kinase-3β (GSK-3β), p-PI3K, PI3K, p-AKT, AKT, and β-actin at 4°C overnight. Then the PVDF membrane was incubated with horseradish peroxidase (HRP)-conjugated secondary antibody (diluted with 5% skimmed milk to 1:2,000) for 1 h at room temperature. Proteins were detected using a chemiluminescence assay. The antigen–antibody complex was visualized using a HRP substrate (Millipore, Billerica, MA, USA) and the ChemiScope 5300 Pro CCD camera (Clinx Science Instruments, Shanghai, China). The band intensity quantification was analyzed by Quantity One software (Bio-Rad, CA, USA). The primary antibodies of c-Myc (#5605), cyclin D1 (#2978), p-PI3K (#4228), PI3K (#4292), p-AKT (#4060), AKT (#4691), and β-actin (#4970) were purchased from Cell Signaling Technology. The β-catenin primary antibody (#ab32572) was purchased from Abcam (UK), and the GSK-3β primary antibody (#A2081) was purchased from ABclonal (China).

### Immunofluorescence Staining

The cells were seeded on a 24-well cell culture plate and were treated with LPS, MEL (0.5 μM), or LPS–MEL (0.5 and 5 μM) for 15 min. Then the cells were fixed with 4% paraformaldehyde for 15 min. After washing with PBS, the membrane was penetrated with 0.4% Triton X-100 (ST797, Beyotime, China) for 15 min. The cells were washed with PBS three times and were blocked by 5% BSA for 1.5 h at room temperature. The cells were incubated with anti-β-catenin (all at 1:250 in 5% BSA) at 4°C overnight. After PBS washings for three times, the cells were incubated with an FITC-conjugated secondary antibody (A0423, Beyotime, China) for 1.5 h at room temperature. The nuclei were stained with DAPI (C1005, Beyotime, China). Finally, the cells were visualized with a fluorescence microscope (Leica TCS SP8, Leica Corporation, Germany).

### Statistical Analysis

The experiment was repeated at least three times. All the data were analyzed using SPSS 21.0 software (IBM, NY, USA). Statistically significant differences throughout this study were calculated by one-way ANOVA, followed by Dunnett's test. The data were shown as means ± standard error of means (SEM). A bilateral *P* < 0.05 was considered statistically significant.

## Results

### BEEC Viability

**Figure 2:**
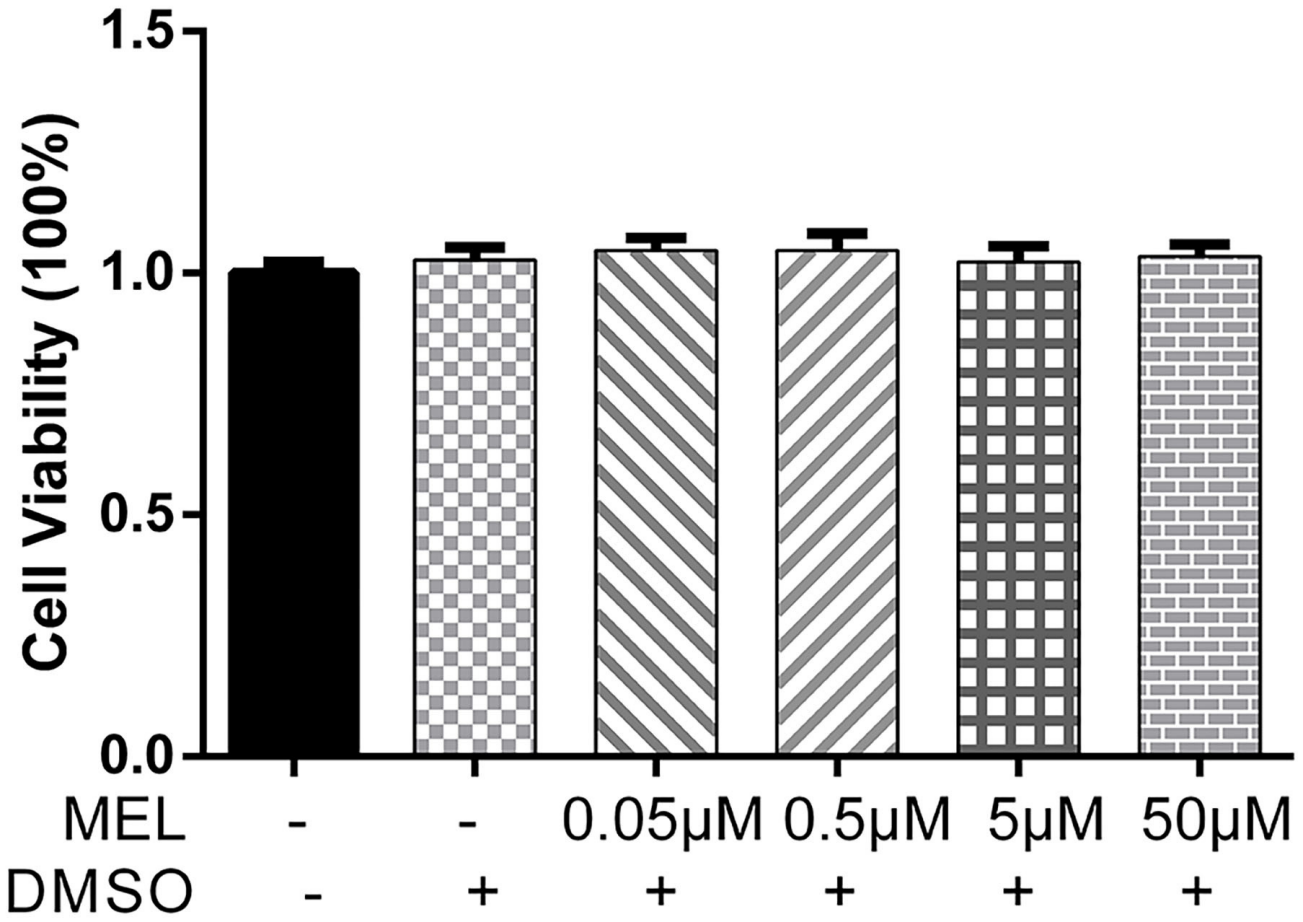
The effect of meloxicam on the viability of primary bovine endometrial epithelial cells. MEL, meloxicam. The cells were treated with MEL (5 × 10^−2^, 5 × 10^−1^, 5, and 50 μM) alone for 24 h. The cell viability was detected using the Cell Counting Kit-8 method. All data were presented as means ± SEM (*n* = 3).

**Figure 3:**
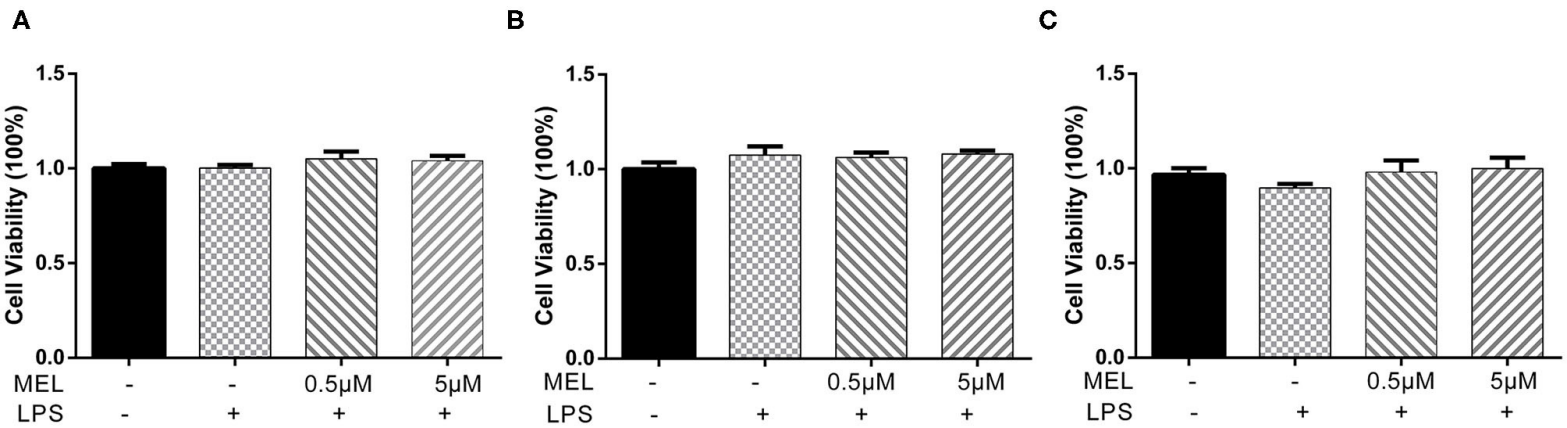
The effect of meloxicam on the viability of primary bovine endometrial epithelial cells stimulated with lipopolysaccharide. LPS, lipopolysaccharide; MEL, meloxicam. The cells were co-treated with MEL (0.5 and 5 μM) and LPS (10 μg/ml) for 24 **(A)**, 48 **(B)**, and 72 h **(C)**. All data were presented as means ± SEM (*n* = 3).

The CCK-8 method was used to detect the effect of MEL on BEEC viability. The viability of BEEC was not influenced (*P* > 0.05) by various concentrations (5 × 10^−2^, 5 × 10^−1^, 5, and 5 × 10^1^ μM) of MEL (Figure 2). As shown in Figure 3, no difference (*P* > 0.05) was observed in the cell viability between the cells treated with only LPS and the cells co-treated with LPS and MEL (0.5 and 5 μM).

### BEEC Scratch Test

**Figure 4:**
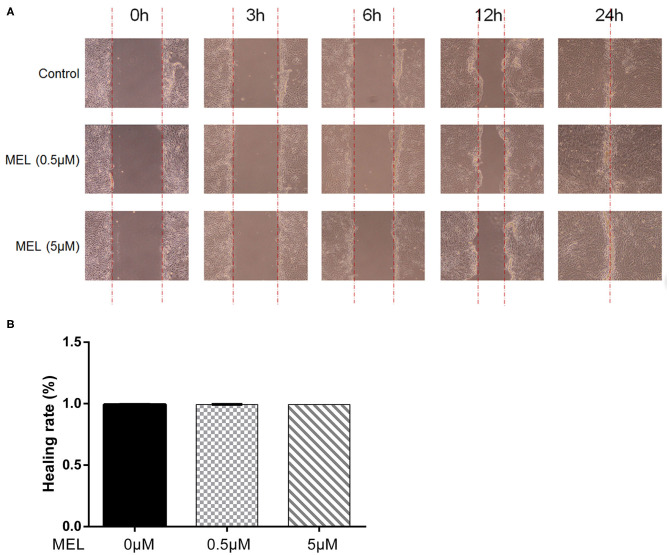
The effect of meloxicam on the healing rate of bovine endometrial epithelial cells using the cell scratching test. MEL, meloxicam. **(A)** The cells were treated with 0.5 and 5 μM MEL for 0, 3, 6, 12, and 24 h to observe the cell healing under light microscopy at ×100 magnification. The parallel red dotted lines indicate the cell margins in the control group at each time point. **(B)** The healing rate of cells was detected at 24 h using Image Pro Plus 6.0 software. Healing rate = (scratch width at 0 h – scratch width at 24 h)/scratch width at 0 h × 100%. All data were presented as means ± SEM (*n* = 3).

**Figure 5:**
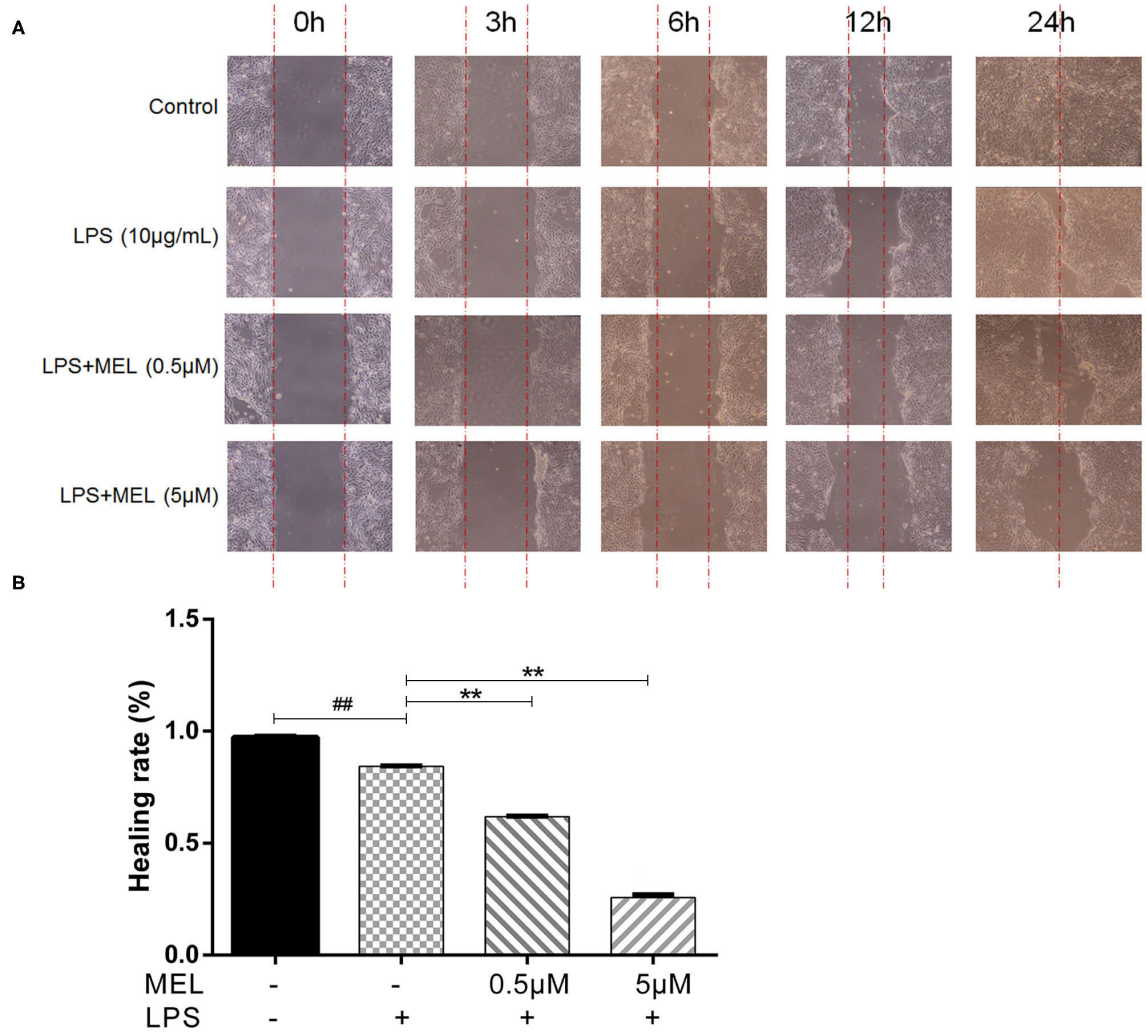
The effect of meloxicam on the healing rate of lipopolysaccharide-stimulated bovine endometrial epithelial cells using the cell scratch test. LPS, lipopolysaccharide; MEL, meloxicam. **(A)** The cells were treated with LPS or co-treated with LPS and MEL (0.5 and 5 μM) for 0, 3, 6, 12, and 24 h to observe the cell healing under light microscopy at ×100 magnification. The parallel red dotted lines indicate the cell margins in the control group at each time point. **(B)** The healing rate of cells was detected at 24 h using Image-Pro Plus software 6.0. Scratch width = scratch area/scratch length; Healing rate = (scratch width at 0 h – scratch width at 24 h)/scratch width at 0 h × 100%. #*P* < 0.05, difference compared with the control; ##*P* < 0.01, difference compared with the control; *P < 0.05, difference compared with the LPS treatment group; **P < 0.01, difference compared with the LPS treatment group. All data were presented as means ± SEM (*n* = 3).

The effect of MEL on the healing rate of BEEC was detected using the scratching test. There was no difference (*P* > 0.05) in cell migration at 24 h in cells treated with 0.5 or 5 μM MEL alone (Figure 4). The cell healing rate of the LPS group was lower (*P* < 0.01) than that of the blank control group. Compared with the LPS group, the cell healing rate decreased (*P* < 0.01) in the LPS–MEL groups (Figure 5).

### BEEC Cycle

**Figure 6:**
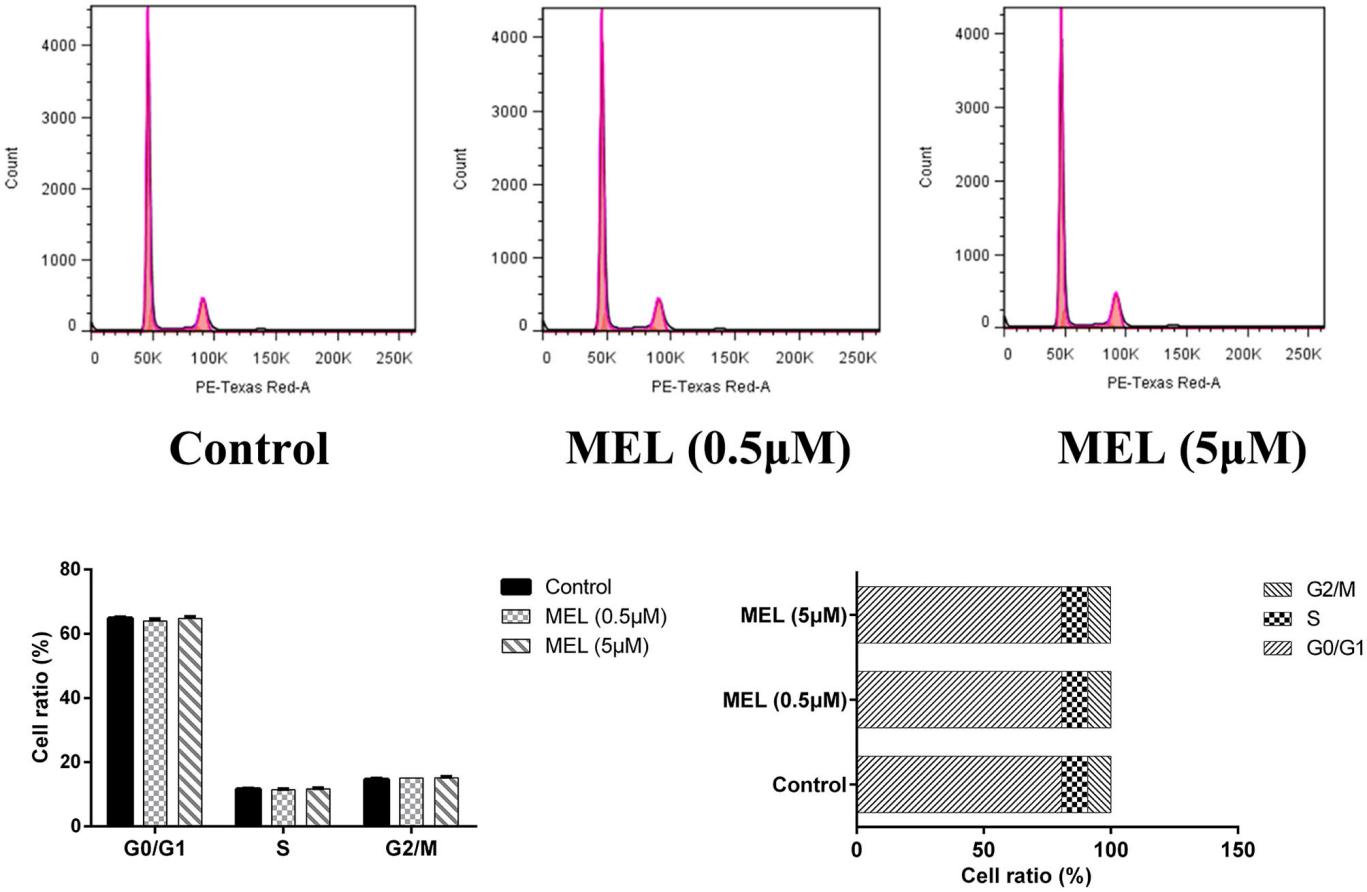
The effect of meloxicam on the cell cycle distribution of bovine endometrial epithelial cells. MEL, meloxicam. The cells were treated with MEL (0.5 and 5 μM) for 24 h. The cell cycle distribution was detected by flow cytometry and was analyzed by FlowJo software 7.6 to obtain the relative number of cells in each phase of the cell cycle. All data were presented as means ± SEM (*n* = 3).

**Figure 7:**
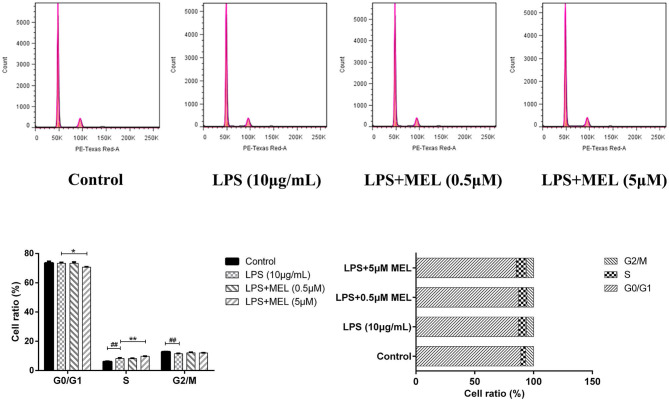
The effect of meloxicam on the cell cycle distribution of bovine endometrial epithelial cells stimulated with lipopolysaccharide. LPS, lipopolysaccharide; MEL, meloxicam. The cells were treated with LPS or co-treated with LPS and MEL (0.5 and 5 μM) for 24 h. The cell cycle distribution was detected by flow cytometry and was analyzed by FlowJo software 7.6 to obtain the relative number of cells in each phase of the cell cycle. #*P* < 0.05, difference compared with the control; ##*P* < 0.01, difference compared with the control; *P < 0.05, difference compared with the LPS treatment group; **P < 0.01, difference compared with the LPS treatment group. All data were presented as means ± SEM (*n* = 3).

The influence of MEL on the cell cycle of BEEC was determined using flow cytometry. As shown in Figure 6, MEL had no effect (*P* > 0.05) on the number of cells in each cell cycle. After treatment with LPS for 24 h, the number of cells increased (*P* < 0.01) in the S phase and decreased (*P* < 0.01) in the G2 phase, indicating the cell cycle arrest in the S phase. After treatment with LPS and MEL for 24 h, the number of cells decreased (*P* < 0.01) in the G1 phase and increased (*P* < 0.01) in the S phase, suggesting the cell cycle arrest in the G0/G1 phase (Figure 7).

### Gene Expressions of *PTGS1, PTGS2, TLR4*, and Growth Factors in BEECs

**Figure 8:**
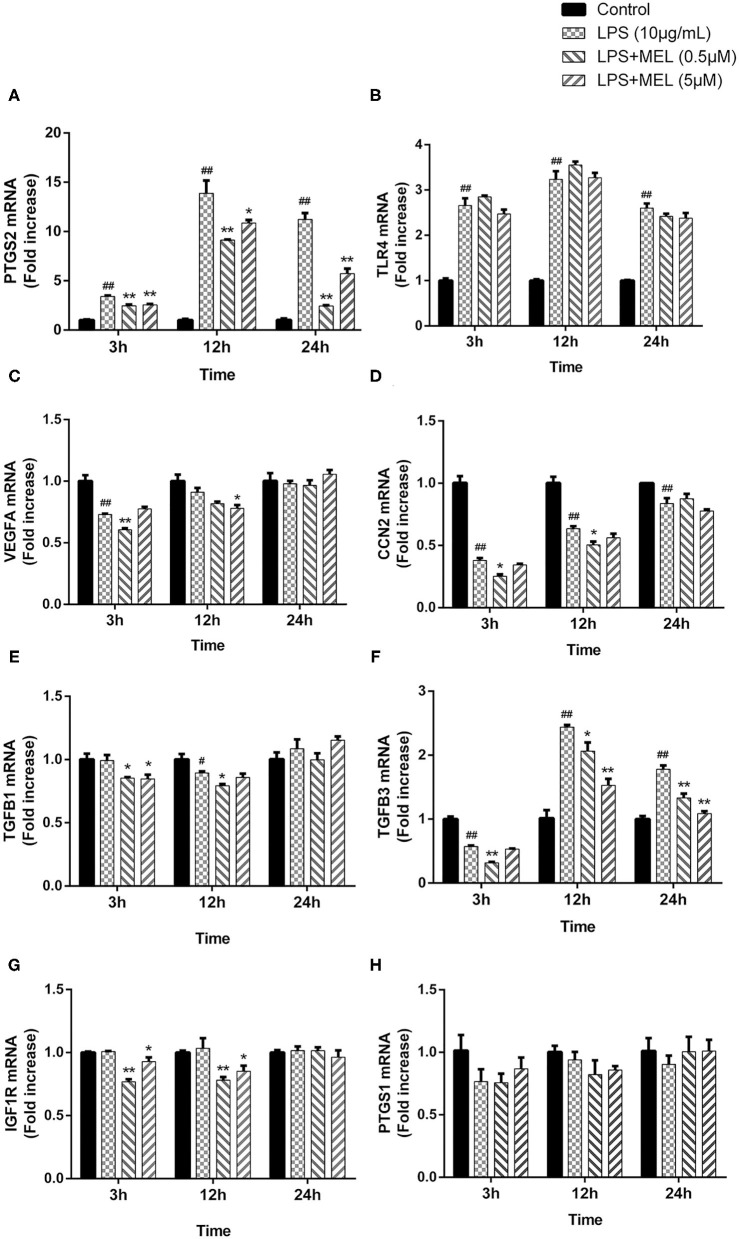
The effect of meloxicam on the mRNA expressions of *PTGS2* **(A)**, *TLR4* **(B)**, *VEGFA* **(C)**, *CCN2* **(D)**, *TGFB1* **(E)**, *TGFB3* **(F)**, *IGF1R* **(G)**, and *PTGS1* **(H)** in bovine endometrial epithelial cells stimulated with lipopolysaccharide. LPS, lipopolysaccharide; MEL, meloxicam. The cells were treated with LPS or co-treated with LPS and MEL (0.5 and 5 μM) for 3, 12, or 24 h. The relative mRNA abundance was detected by quantitative reverse-transcription polymerase chain reaction and was analyzed by the 2^−ΔΔCT^ method. *ACTB* was used as the housekeeping gene. Each group was repeated three times. #*P* < 0.05, difference compared with the control; ##*P* < 0.01, difference compared with the control; *P < 0.05, difference compared with the LPS treatment group; **P < 0.01, difference compared with the LPS treatment group. All data were presented as means ± SEM (*n* = 3).

The effects of MEL on the gene expressions of *PTGS1, PTGS2, TLR4*, and the growth factors (*VEGFA, CCN2, IGF1R, TGFB1*, and *TGFB3*) were detected using quantitative reverse-transcription PCR. As shown in Figures 8A,B, compared with the blank control, the gene expressions of *PTGS2* and *TLR4* increased (*P* < 0.01) after LPS stimulation at 3, 12, and 24 h. Compared with the LPS group, the *PTGS2* gene expression decreased (*P* < 0.01) in LPS–MEL groups. There was no difference (*P* > 0.05) in the *TLR4* transcription between the LPS group and the LPS–MEL groups. Compared with the blank control group, the gene expression of most growth factors showed no change (*P* > 0.05) or a downregulation (*P* < 0.05) after LPS treatment at observed time points, except *TGFB3* at 12 and 24 h (Figures 8C–G). The gene expression of growth factors in LPS–MEL groups were generally lower (*P* < 0.05) than those in the LPS group at 3 and 12 h. Other than the decreased mRNA abundance of *TGFB3* (*P* < 0.05) in the LPS–MEL groups as compared with the LPS group, we found no other differences (*P* > 0.05) at 24 h. The mRNA abundance of *PTGS1* was not influenced (*P* > 0.05) by LPS or LPS–MEL treatment (Figure 8H).

### Wnt/β-Catenin Activation in BEECs

**Figure 9:**
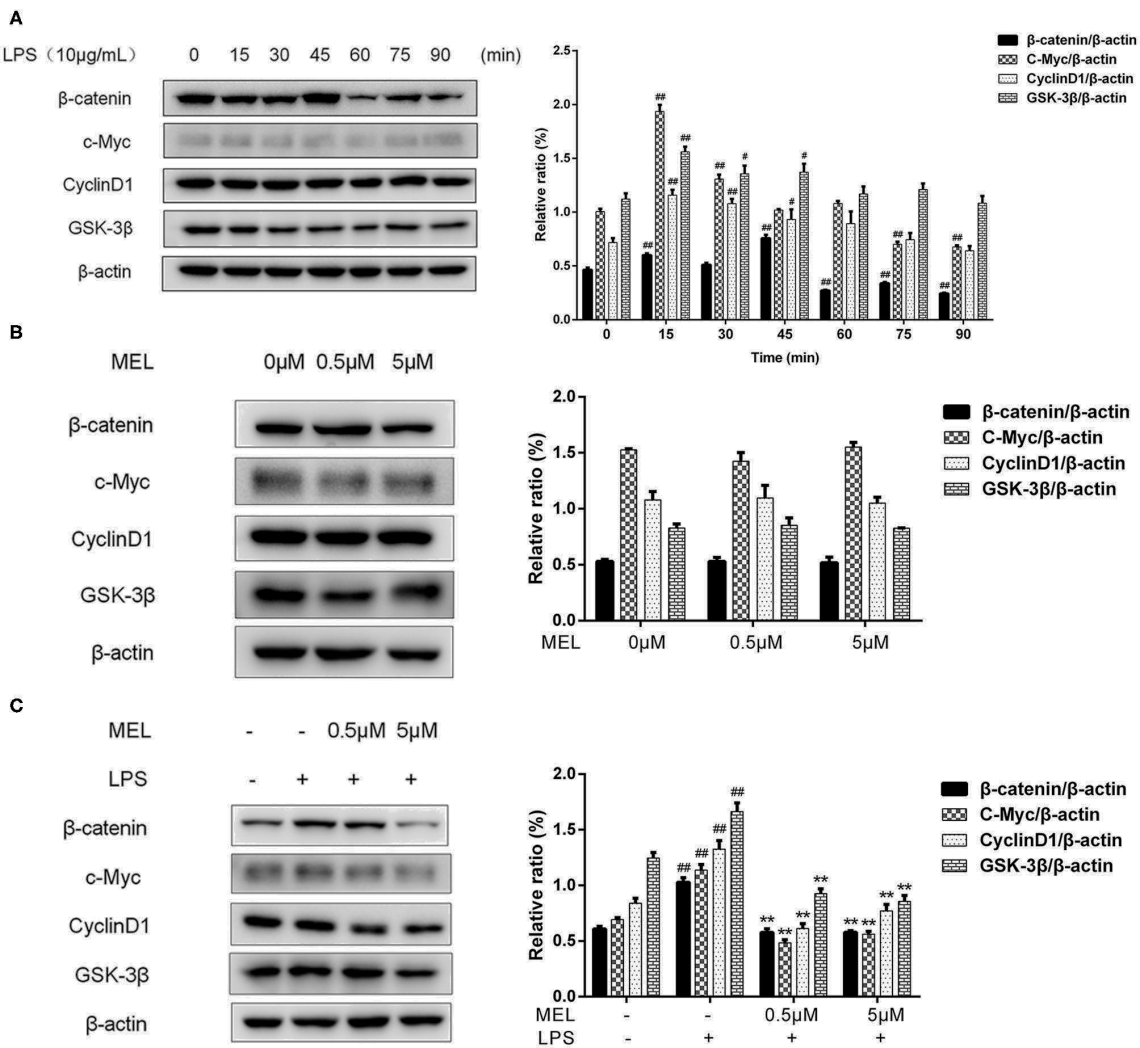
The effect of meloxicam on the Wnt/β-catenin pathway in bovine endometrial epithelial cells. The protein levels of β-catenin, c-Myc, cyclin D1, and GSK-3β were determined using western blot. The band intensity quantification was analyzed by Quantity One software. β-Actin was used as the internal control. LPS, lipopolysaccharide; MEL, meloxicam. **(A)** The cells were treated with LPS for 0, 15, 30, 45, 60, 75, and 90 min. **(B)** The cells were treated with MEL alone for 15 min. **(C)** The cells were treated with LPS or co-treated with LPS and MEL (0.5 and 5 μM) for 15 min. #*P* < 0.05, difference compared with the control; ##*P* < 0.01, difference compared with the control; *P < 0.05, difference compared with the LPS treatment group; **P < 0.01, difference compared with the LPS treatment group. All data were presented as means ± SEM (*n* = 3).

The effects of MEL on the protein levels of β-catenin, c-Myc, cyclin D1, and GSK-3β were detected using western blot. As shown in Figure 9A, the protein level of β-catenin increased (*P* < 0.01) at 15 and 45 min and decreased (*P* < 0.01) at 60, 75, and 90 min. The levels of c-Myc and cyclin D1 increased (*P* < 0.05) at 15 and 30 min. The level of GSK-3β increased (*P* < 0.05) at 15, 30, and 45 min. Based on these results, we selected 15 min to detect the changes in the Wnt/β-catenin pathway in subsequent experiments.

MEL (0.5 and 5 μM) treatment alone did not influence (*P* > 0.05) the protein levels of β-catenin, c-Myc, cyclin D1, or GSK-3β as compared with the blank control (Figure 9B). LPS stimulation caused an increase (*P* < 0.01) in the levels of β-catenin, c-Myc, cyclin D1, and GSK-3β (Figure 9C). Compared with the LPS group, the levels of these proteins were lower (*P* < 0.01) in the LPS–MEL groups.

### β-Catenin Distribution in BEECs

**Figure 10:**
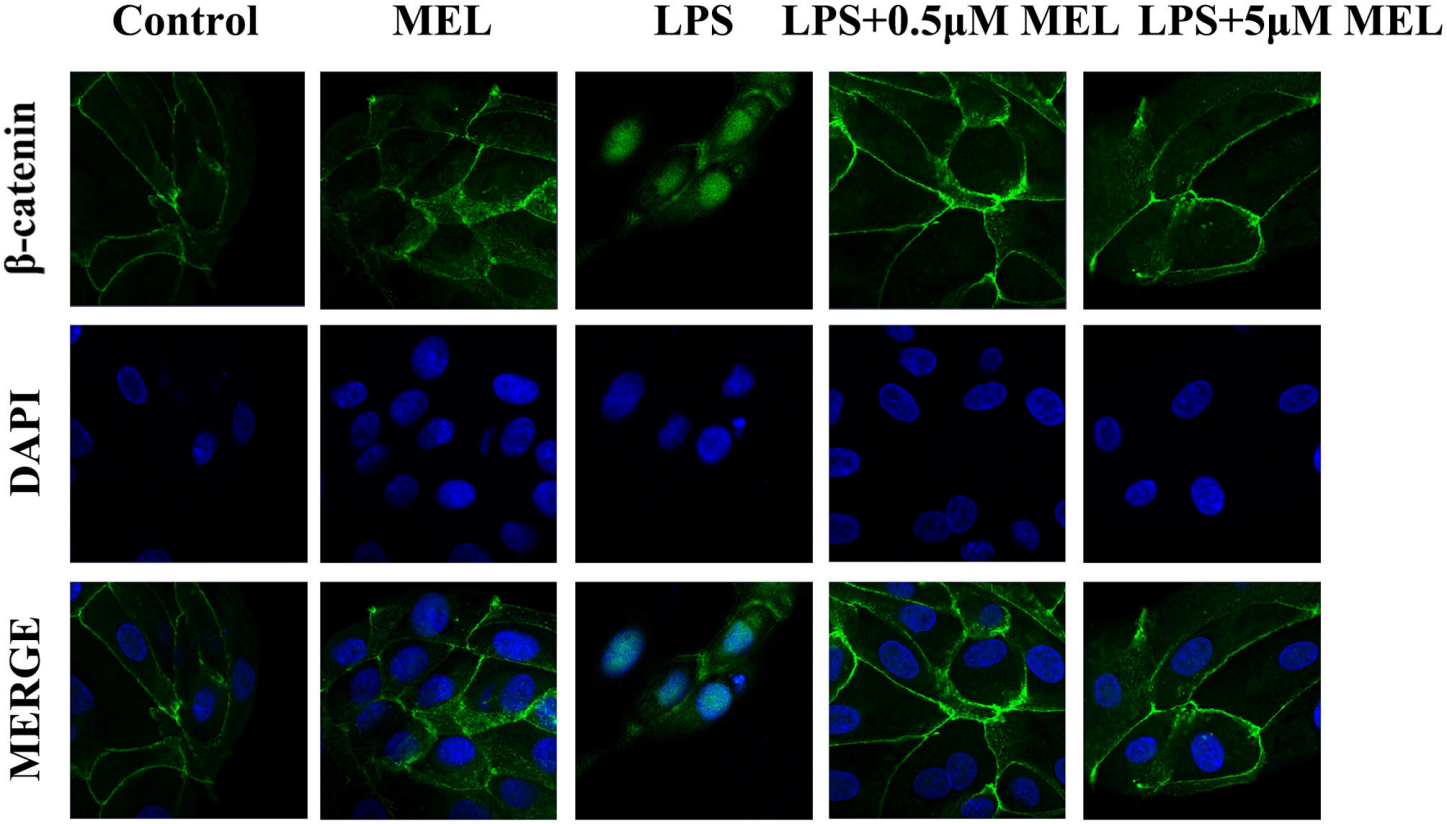
The effect of meloxicam on Wnt/β-catenin activation in bovine endometrial epithelial cells. LPS, lipopolysaccharide; MEL, meloxicam. The cells were treated with LPS or MEL (0.5 μM) or co-treated with LPS and MEL (0.5 and 5 μM) for 15 min. The β-catenin were observed by confocal microscopy. All data were presented as means ± SEM (*n* = 3).

As shown in Figure 10, the β-catenin level on the cytomembrane in the MEL (0.5 μM) group was similar to that in the control group. In the LPS group, an increased number of β-catenin was observed to enter the nucleus. Compared with the LPS group, the amount of β-catenin in the nucleus in LPS–MEL groups seemed lower, and the β-catenin was mainly detected in the cytomembrane.

### PI3K/AKT Activation in BEECs

**Figure 11:**
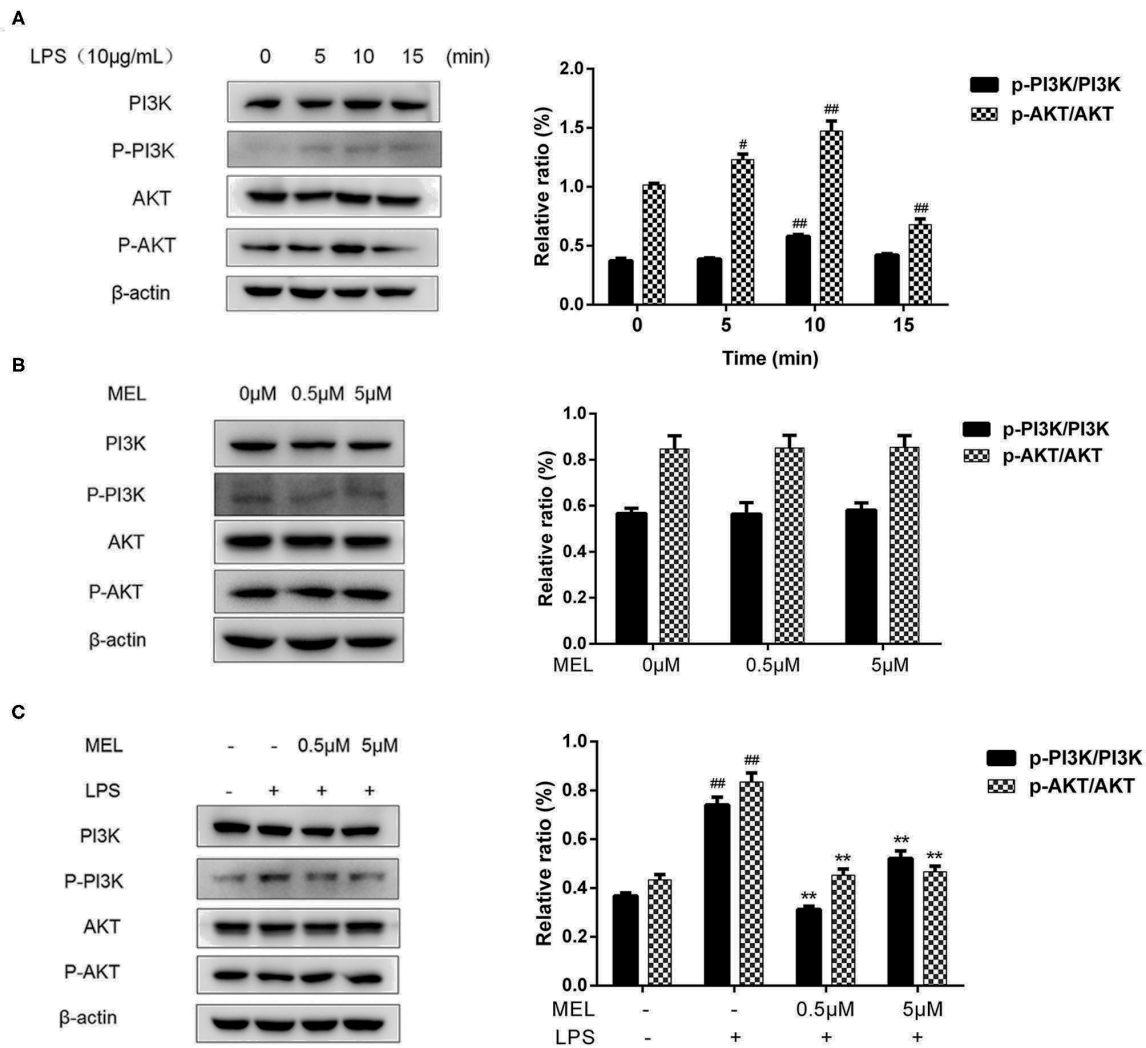
The effect of meloxicam on PI3K and AKT phosphorylations in bovine endometrial epithelial cells. LPS, lipopolysaccharide; MEL, meloxicam. The p-PI3K, PI3K, p-AKT, and AKT levels were determined using western blot. The band intensity quantification was analyzed by Quantity One software. β-Actin was used as the internal control. **(A)** The cells were treated with LPS for 0, 5, 10, and 15 min. **(B)** The cells were treated with MEL (0.5 and 5 μM) for 10 min. **(C)** The cells were treated with LPS or co-treated with LPS and MEL (0.5 and 5 μM) for 10 min. #*P* < 0.05, difference compared with the control; ##*P* < 0.01, difference compared with the control; *P < 0.05, difference compared with the LPS treatment group; **P < 0.01, difference compared with the LPS treatment group. All data were presented as means ± SEM (*n* = 3).

The influence of MEL on the PI3K/AKT pathway was detected using western blot and is depicted in Figure 11A. The phosphorylation levels of PI3K and AKT showed similar trends. The p-PI3K/PI3K ratio increased (*P* < 0.01) at 10 min, whereas the of p-AKT/AKT ratio increased (*P* < 0.05) at 5 and 10 min and decreased at 15 min. Based on these results, the time point of 10 min was selected for subsequent experiments.

MEL alone (0.5 or 5 μM) showed no effect (*P* > 0.05) on the PI3K/AKT pathway (Figure 11B). Compared with the blank control group, the phosphorylation levels of PI3K and AKT increased (*P* < 0.01) in the LPS group. Compared with the LPS group, the phosphorylation levels of PI3K and AKT decreased (*P* < 0.01) in the LPS–MEL groups (Figure 11C).

## Discussion

The COX-1 and COX-2 were encoded by *PTGS1* and *PTGS2* genes, respectively. In this study, MEL reduced the *PTGS2* but not *PTGS1* transcription in BEEC stimulated with LPS, implying its preferential inhibitory effect on COX-2 over COX-1. MEL inhibited PI3K/AKT and Wnt/β-catenin pathways and the downstream c-Myc, cyclin D1, and VEGF, resulting in the cell cycle block and the downregulation of cell proliferation in LPS-stimulated BEEC. This was similar to the report that the cell survival was inhibited by MEL treatment in LPS-challenged bovine mammary epithelial cells (20). These results indicated that MEL treatment may induce tissue damage in the endometrium. Whether the character of this discovery would support or impair the elimination of bacteria from infected uterine cavity during metritis or endometritis therapy with MEL still needs a comprehensive investigation.

The results showed that 0.5 and 5 μM MEL with or without 10 μg/ml LPS treatment had no effect on BEEC viability. By observing the cell scratch test, MEL alone had no effect on cell migration. This was consistent with the results of Goncalves et al. (27), showing that 5 μM MEL had no effect on cell viability and migration rate in adult mouse brain neural cells. When BEEC was co-treated with MEL and LPS, the cell healing rate reduced. Similarly, Chechik et al. (28) demonstrated that MEL interfered with the normal healing process of the raptured supraspinatus tendon in rats, which was supported by Cohen et al. (29), who found that celecoxib inhibited tendon-to-bone healing, and by Ferry et al. (30), who reported that ibuprofen had a detrimental effect on healing strength at the bone–tendon junction. The discrepancy between the result of MEL treatment alone and LPS-MEL suggested that the action of MEL on BEEC proliferation was indirect.

COX-2 is a pathological inducer, which catalyzes the synthesis of PG and participates in inflammatory reaction. Our results showed that MEL inhibited LPS-induced *PTGS2* transcription in BEEC, and many studies supported this result (31, 32). It has been reported that TLR4 mainly exists in bovine endometrial cells (6). The recognition of LPS by TLR4 activates the nuclear factor kappa-B (NF-κB) pathway, which promotes the transcription of proinflammatory cytokines, causing inflammatory response (33). Our results were consistent with reports that LPS promoted *TLR4* gene expression. However, MEL did not attenuate the LPS-induced *TLR4* gene expression, which may be explained by its effect on the downstream NF-κB pathway (34). Growth factors (VEGF, CTGF, IGFR, TGF-β1, and TGF-β3) play a regulatory role in the proliferation, differentiation, matrix repair, and remodeling of endometrial epithelial cells and stromal cells (8, 35, 36). Our result showed that MEL inhibited gene expressions of *VEGFA, CCN2, IGF1R, TGFB1*, and *TGFB3* in LPS-stimulated BEEC to varying degrees. VEGF and CTGF were encoded by *VEGFA* and *CCN2* genes, respectively. Quintana et al. found that MEL inhibited VEGF expression in ovarian hyperstimulation syndrome (37). Other studies have found that N-(2-cyclohexyloxy-4-nitrophenyl)-methanesulfonamide, another preferential inhibitor of COX-2 over COX-1, reduced the human pancreatic cancer cell number through inhibiting the expressions of VEGF and CTGF (38). Tolfenamic acid has been shown to inhibit the proliferation of hepatoma cells by regulating TGF-β1 expression (39). These reports supported the results of our experiment that MEL downregulated the mRNA abundance of *VEGFA, CCN2, IGF1R, TGFB1*, and *TGFB3* and inhibited the proliferation and growth of BEEC.

Wnt/β-catenin is involved in wound healing, uterine development, embryo implantation, and uterine involution (40, 41). Cheng et al. found that LPS activated the Wnt/β-catenin pathway in a rat model of pneumonia (42). Similarly, we found that the levels of β-catenin, c-Myc, cyclin D1, and GSK-3β increased after LPS stimulation. NSAIDs have been shown to inhibit GSK-3β protein expression and to downregulate the transcription of β-catenin/TCF responsive gene (43, 44). Vallee et al. (45) demonstrated that NSAIDs inhibited the Wnt/β-catenin pathway and alleviated inflammatory response. In this study, LPS–MEL decreased the levels of β-catenin, GSK-3β, c-Myc, and cyclin D1 and prevented β-catenin from entering the nucleus, indicating the inhibition of the Wnt/β-catenin pathway by MEL. Cyclin D1 and c-Myc are the downstream factors of the Wnt/β-catenin pathway and are essential for cell cycle progression (46). Our results showed that the cell cycle was arrested in the G0/G1 phase in LPS–MEL groups. In cancer studies, NSAIDs could induce cell cycle arrest, apoptosis, and angiogenesis inhibition (47). Arantes-Rodrigues et al. found that MEL reduced the proliferation of bladder cancer cells and arrested the cells in the G0/G1 phase (48). These results revealed that MEL blocked the cell cycle of BEEC through the Wnt/β-catenin pathway and its downstream cyclin D1 and c-Myc. In addition, TGF-β1 has been shown to activate the Wnt/β-catenin pathway (49). Our results showed that LPS-MEL reduced the gene expression of *TGFB1*, suggesting another potential target of MEL in inhibiting the Wnt/β-catenin pathway.

The PI3K/AKT pathway regulates various cellular processes such as cell proliferation and differentiation (50, 51). AKT, mainly activated by PI3K, induces cell proliferation and survival (52). In accordance with previous report, we found that the phosphorylation of PI3K and AKT increased after LPS stimulation (53). Evidence has shown that NSAID inhibits the AKT signaling pathway. In the treatment of endometrial cancer, MEL has played a role through the PI3K/AKT pathway (54). Similarly, our results indicated the inhibitory effect of MEL on LPS-induced PI3K/AKT activation. Wnt/β-catenin and PI3K/AKT pathways are interconnected by intracellular GSK-3β. The phosphorylated form of AKT activates GSK-3β, causing the adenomatous polyposis coli/axin/GSK-3β complex to lose its binding ability to β-catenin, allowing β-catenin to enter the nucleus (55). NSAIDs induce GSK-3β phosphorylation and inhibit the phosphorylation of PI3K and AKT, so that GSK-3β continues to be downregulated at the phosphorylation level, and the negative feedback on PI3K/AKT pathway has a more obvious inhibitory effect (56). Moreover, IGFR has been shown to activate the PI3K/AKT pathway (57); the decreased gene expression of *IGF1R* in the LPS–MEL groups may further inhibit the activation of the PI3K/AKT pathway. Further studies are required to clarify the mechanism.

In conclusion, MEL reduced the cell proliferation, blocked the cell cycle progression, and inhibited the gene expressions of *VEGFA, CCN2, IGF1R, TGFB1*, and *TGFB3* in LPS-challenged BEEC. This inhibitory effect of MEL was possibly mediated by Wnt/β-catenin and PI3K/AKT pathways.

## Data Availability Statement

The original contributions presented in the study are included in the article/Supplementary Material, further inquiries can be directed to the corresponding author/s.

## Author Contributions

LC: conceptualization and methodology. YQ: data curation, writing—original draft preparation, and software. HC: visualization, software, and validation. JD and JuL: investigation. HW and CQ: supervision. JiL: supervision, writing—reviewing, and editing. All authors contributed to the article and approved the submitted version.

## Conflict of Interest

The authors declare that the research was conducted in the absence of any commercial or financial relationships that could be construed as a potential conflict of interest.
